# A 14-Year Follow-Up of a Combined Liver-Pancreas-Kidney Transplantation: Case Report and Literature Review

**DOI:** 10.3389/fmed.2020.00148

**Published:** 2020-04-28

**Authors:** Geng Zhang, Weijun Qin, Jianlin Yuan, Changsheng Ming, Shuqiang Yue, Zhengcai Liu, Lei Yu, Ming Yu, Xiaokang Gao, Yu Zhou, Longxin Wang, Xiaojian Yang, Kefeng Dou, He Wang

**Affiliations:** ^1^Department of Urology, Xijing Hospital, Fourth Military Medical University, Xi'an, China; ^2^Institute of Organ Transplantation, Tongji Hospital, Tongji Medical College, Huazhong University of Science and Technology, Wuhan, China; ^3^Department of Hepatobiliary, Xijing Hospital, Fourth Military Medical University, Xi'an, China; ^4^Department of Ultrasound Diagnostics, Xijing Hospital, Fourth Military Medical University, Xi'an, China; ^5^Department of Urology, General Hospital of Xinjiang Military Region, Urumqi, China; ^6^Department of Urology, Central Theater Command General Hospital of The Chinese People's Liberation Army, Wuhan, China; ^7^Urology Department, Maanshan People's Hospital, Maanshan, China

**Keywords:** liver, pancreas, renal, simultaneous transplantation, diabetes mellitus

## Abstract

**Objective:** To investigate the long-term effect of triple organ transplantation (liver, kidney, and pancreas) in a patient with end-stage liver disease, post chronic hepatitis B, cirrhosis, chronic renal failure, and insulin-dependent diabetes mellitus caused by chronic pancreatitis and to explore the optimal surgical procedure.

**Case:** A 43-year-old man with progressive emaciation and hypourocrinia for 2 months. Results indicated exocrine pancreatic insufficiency and insulin-dependent diabetes related to chronic pancreatitis (CP) after developing end-stage hepatic and renal failure. Simultaneous piggyback orthotopic liver and heterotopic pancreas-duodenum and renal transplantation was performed in 2005. Pancreatic exocrine secretions were drained enterically to the jejunum, and the donor kidney was placed in the left iliac fossa. Patient was prescribed with prednisone, tacrolimus, mycophenolate mofetil, Rabbit Anti-human Thymocyte Immunoglobulin, and simulect for immunosuppression.

**Results:** Satisfactory hepatic and pancreatic functional recovery was achieved within 7 days post-surgery. The kidney was not functional, and continuous renal replacement therapy was used. However, the donor kidney was removed at day 16 post-surgery due to acute rejection reaction. A new renal transplantation at the same position was performed, and satisfactory kidney function from the new graft was achieved 3 days later. In 14 years of follow-up, patient has not had any rejection reactions or other complications such as pancreatitis, thrombosis, and localized infections. The patient is insulin independent with normal liver and renal functions. FK506+Pred was used for immunosuppression, and the tac tough level maintained 3.0–4.5 ng/ml. Lamivudine was prescribed for long-term use to inhibit HBV virus duplication.

**Conclusion:** Simultaneous piggyback orthotopic liver and heterotopic pancreas-duodenum and renal transplantation is a good therapeutic option for patients with exocrine pancreatic insufficiency and insulin-dependent diabetes combined with hepatic and renal failure.

## Background

On January 17, 2005, we performed a pancreatic jejunal drainage and *in situ* piggyback type combined liver-pancreas-kidney transplantation in a patient with post-hepatitis B cirrhosis, hepatic insufficiency insulin, chronic renal insufficiency, accompanied dependent diabetes mellitus caused by chronic pancreatitis in our hospital. The patient has been followed up for more than 14 years and is the longest survivors of similar operations in the world. The follow-up information is reported as follows.

A 43-year-old man was detected positive for hepatitis B surface antigen in 1994, but was not followed-up regularly. From October 2004, progressive weight loss and decreased urine output was noted and the patient was admitted to the hospital on November 20, 2004. By January 2005, patient's body weight reduced by 15 kg, and preoperative body mass was 60.5 kg. Physical examination identified discomfort in the right upper abdomen and abdominal distension. Laboratory examination was as follows. Blood routine: white blood cells (WBC) 7.2 × 10^9^/L, red blood cells (RBC) 3.4 × 10^12^/L, Hb 6 g/L, PLT 70 × 10^12^/L. Urine routine: protein +++, occult blood ++. Liver function: alanine aminotransferase (ALT) 117 U/L, aspartate aminotransferase (AST) 113 U/L, total protein (TP) 50 g/L, albumin (ALB) 26.9 g/L, alkaline phosphatase (ALP) 99 U/L, γ-glutamyltranspeptidase (GGT) 109 U/L, total bilirubin (TBIL) 102 μmol/L. Renal function: blood urea nitrogen (BUN) 23.6 mmol/L, creatinine (CRE) 664 μmol / L. Hepatitis B series tips: hepatitis B surface antigen (HBsAg), hepatitis B e antibody (HBeAb), hepatitis B core antibody (anti-HBC) positive, HBV-DNA 1.5 × 10^7^/ml. Ascites was yellow turbid with RBC 2,200 × 10^6^/ml, WBC 50 × 10^6^/ml, GLU 9.5 mmol/L, TP 9 g/L, ALB 6.0 g/L. Fasting and postprandial blood sugar were 10.8 and 18.4 mmol/L, respectively. Ultrasonography showed cirrhosis, a large amount of ascites, splenomegaly, large pancreatic head, and expansion of the main pancreatic duct. CT examination showed cirrhosis, ascites, portal hypertension, cholecystitis, atrophy of the pancreatic body and tail, significant expansion of the pancreatic duct, full pancreatic head, and atrophy of both kidneys. Magnetic resonance imaging showed cirrhosis with moderate ascites, obvious pancreatic duct dilatation, bilateral kidney atrophy, and cholecystitis. Renal dynamic imaging showed severe damage to both kidneys. The non-functional left and right renal glomerular filtration rate (GFR) were ~3.73 ml/min/1.73 m^2^ and 5.46 ml/min/1.73 m^2^, respectively. The patient was diagnosed with post-hepatitis B cirrhosis, hepatic insufficiency with chronic renal insufficiency and chronic pancreatitis leading to insulin-dependent diabetes mellitus (IDDM). A simultaneous liver-pancreas-kidney transplantation surgery was planned.

The recipient blood type was type B, Rh+. The donor blood types were O-type and B-type. The panel reactive antibody (PRA) was negative for the recipient. The human leukocyte antigen (HLA) sites of the donors and recipient are shown in Table 1. Both pre-transplant lymphotoxicity tests were negative.

**Table 1:** Basic characteristics of the recipient and donors.

**Recipient/donor**	**Gender**	**Age**	**Source of donors**	**Organ types**	**HLA sites**					
Recipient	Male	43	—	—	A2	A33	B52	B54	DR9	DR13
Donor 1	Male	40	DCD	Liver; pancreas; right kidney	A2	A24	B15	B46	DR9	DR53
Donor 2	Male	52	DCD	Right kidney	A9	A28	B17	B50	DR4	DR11

*DCD, donation after citizen's death; HLA, human leukocyte antigen*.

**Figure 1:**
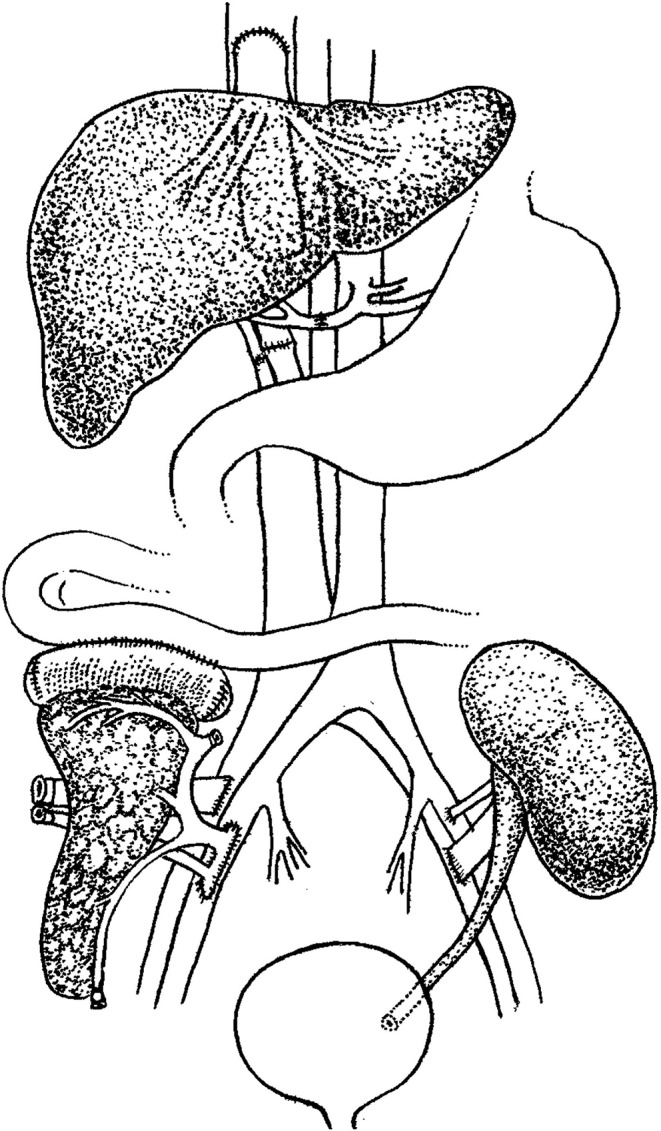
Schematic diagram of the combined liver-pancreas-kidney transplantation. Transplantation was performed in the order of liver, kidney, and pancreas with piggyback orthotopic liver transplantation, conventional renal transplantation, and jejunal drainage of pancreas transplantation, respectively.

In the initial operation, the organs were obtained from donor 1 and trimmed using routine methods (1), and transplanted in the order of liver, kidney, and pancreas. Under general anesthesia, an incision under the bilateral costal margin was made, and a piggyback orthotopic liver transplantation was performed. Further, a conventional kidney transplantation was performed in the left axilla, and the pancreas was implanted into the right axilla. Carrel cuff of the abdominal aorta with superior mesenteric artery and celiac trunk were anastomosed end-to-side with external iliac artery, and portal vein of the transplant pancreas was anastomosed end-to-side with external iliac vein. The donor pancreaticoduodenal segment was anastomosed laterally with the upper jejunum. After anastomosis, the venous and arterial blood supply was opened, the drainage tube was placed, and the incision was sutured (Figure 1).

Following the operation, the liver and kidney function, blood and urine bilirubin, blood sugar, blood and urine routine, blood coagulation, D-dimer, serum C-peptide, blood and urine amylase, tacrolimus (FK506) blood concentration and immune index were monitored. Two doses of basiliximab were used to induction therapy. Three days after operation, acute rejection was considered and rabbit anti-human thymocyte immunoglobulin (ATG) was added, 100 mg/d for 7 days. The immunosuppressive regimen was FK506+ mycophenolate mofetil (MMF) + prednisone acetate (Pred).

One week after operation, ALT 57U/L, AST 43 U/L, ALB 36.9 g/L, TBIL 22 μmol/L, BU 26.5 mmol/L, CRE 683 μmol/L were re-examined. Insulin was completely discontinued. Continuous renal replacement therapy maintained a stable internal environment and blood and urine amylase were normal. Ultrasonography showed that the blood flow of the transplanted hepatic artery, portal vein, inferior vena cava, common bile duct, and pancreatic artery and vein was normal.

On the 16th day after operation, B-mode ultrasonography revealed that the blood flow perfusion of the transplanted kidney decreased significantly, the resistance index of the segmental artery blood flow increased to 1, 24 h urine volume was <300 ml, and PRA was negative. The patient was diagnosed with acute rejection of transplanted kidney. The original transplanted kidney was excised under general anesthesia and a second kidney transplantation (obtained from donor 2) was performed at the original transplanted site 16 days later after diagnosis of rejection of the first kidney. The pathological diagnosis of the original transplanted kidney was acute rejection. The first 24 h urine volume was 7,000 ml after the second operation. On the third day, renal function was reviewed: BUN 6.5 mmol/L, CRE155 μmol/L. One week after the second operation, the patient peripheral blood WBC was 2.1 × 10^9^/L, RBC 1.4 × 10^12^/L, Hb 5.2 g/L, PLT 6 × 10^12^/L, PT 52 s, APTT 120 s, fibrinogen 1.2 g. Surgical wounds continued to ooze blood. Considering the occurrence of bone marrow suppression, MMF was discontinued and immunosuppressive therapy was maintained by FK506 + Pred regimen. The patient was hospitalized for 33 days and discharged after the function of the transplanted liver, kidney, and pancreas was stable.

One month after the operation, the patient returned to a normal diet and resumed social work 3 months later. The patient was regularly monitored for blood routine, liver function, kidney function, eGFR, electrolytes, blood sugar, blood lipids, hepatitis B virus DNA quantification and FK506 blood trough concentration. CT and ultrasound were routinely performed and medication was accordingly adjusted. No clinically visible rejection occurred after the second operation. Hepatitis B check was negative except for positive HBsAb. No other drugs were used except oral immunosuppressive agents, anti-hepatitis B immunoglobulin and lamivudine treatment. By June 2013, physical examination showed that the patient's body weight was 73 kg. Blood routine: WBC 7.6 × 10^9^/L, RBC 4.4 × 10^12^/L, Hb 11.8 g/L, PLT 110 × 10^12^/L. Urine protein and occult blood were negative. Liver function: ALT 20 U/L, AST 19 U/L, TP 62 g/L, ALB 42 g/L, ALP 39 U/L, GGT 38 U/L, TBIL 19 μmol/L. Renal function: BUN 6.5 mmol/L, CRE 130 μmol/L, and blood uric acid 399 μmol/L. Fasting blood glucose was 4.3 mmol/L, postprandial blood glucose was 10.3 mmol/L, and HBV-DNA was <1,000/ml. The amount of FK506 was 1.5 mg twice daily, and the trough concentration was 3.0–4.5 ng/ml. The CT and ultrasound showed that the transplanted liver was *in situ*, the left lobe anterior and posterior diameter was 5.4 cm, and the upper and lower diameter was 3.4 cm, the right lobe anterior and posterior diameter was 11.5 cm, and the upper and lower diameter was 11.5 cm. The morphology was regular, the echo was moderately dense, and the distribution was uniform. The right hepatic artery blood flow velocity Vmax = 55.1 cm/s, RI = 0.71, CDFI showed the color blood flow was well filled. The inner diameter of the portal vein was 0.9 cm, and the blood flow velocity Vmean = 17 cm/s. CDFI showed that the color blood flow was well filled. The inner diameter of the hepatic vein was normal. The transplanted pancreas was located in the right iliac fossa, with a size of 2.7 cm at the head, 1.4 cm at the body, and 2.2 cm at the tail. The echo is moderately dense and evenly distributed. A color blood flow signal can be seen inside. The transplanted kidney was located in the left axilla, the size was 11.8 × 5.3 × 5.3 cm, the boundary was clear, the echo in the parenchyma was dull, the renal pelvis and renal pelvis were separated, and the inner part was a liquid dark area. The maximum anteroposterior diameter was 0.7 cm. The blood flow velocity in the transplanted renal cortex was measured as Vmax = 89.2 cm/s, RI = 0.63. CDFI showed that the color flow was well filled.

## Discussion

### Characteristics of Combined Liver-Pancreas-Kidney Transplantation

Treating end-stage liver disease and IDDM and renal failure with combined liver-pancreas-kidney transplantation is rare because patients with such conditions are rare. There is no inevitable internal relationship among end-stage liver disease, diabetes, and chronic renal failure. Surgical treatment of patients with liver, kidney, and pancreas failure is often difficult because of the patients' poor physical conditions. However, the combined liver-kidney transplantation and pancreas-kidney transplantation are relatively common, and intra- and postoperative management are becoming more advanced. Performing the combined liver-pancreas transplantation is rare, with fewer cases of combined liver-pancreas-kidney transplantation. In addition, the surgical process is complex, and the hemodynamic changes are unstable (2). There are many contradictions regarding the adjustments of medication during and after operation, which increases the difficulty of a successful combined liver-pancreas-kidney transplantation.

Organ transplantation is the best treatment choice for various types of solid organ failure when medical treatment is not effective. Therefore, kidney, liver, and heart transplantations, as well as various types of combined transplantation and organ cluster transplantation, have been performed in various parts of the world. However, because of the poor physical condition of patients, complicated surgical technique, and difficulty in intraoperative and postoperative treatments, the implementation of multiple solid organ combined transplantation is greatly limited.

Surgical indications for combined liver-kidney transplantation are as follows: systemic metabolic diseases such as primary hyperuricemia caused by liver and kidney failure; hereditary diseases such as congenital polycystic liver and polycystic kidney disease, leading to liver and kidney failure; liver and kidney failure caused by different etiologies such as chronic renal failure owing to chronic glomerulonephritis combined with advanced chronic cirrhosis or malignant liver tumors; hepatorenal syndrome; and Wolcott-Rallison syndrome (3). Hepatocirrhosis after viral hepatitis is the main indication of liver transplantation; however, hepatitis B virus reinfection must be prevented early after operation. In our patient, high-dose anti-hepatitis B immunoglobulin was administered to prevent hepatitis B virus infection during and after surgery, and long-term oral lamivudine inhibited the replication of hepatitis B virus. The serum HbsAb titer and hepatitis B virus DNA level were regularly examined, and the dosage of anti-hepatitis B immunoglobulin was adjusted according to the findings (4). Preventing the recurrence of hepatitis B and avoiding the occurrence of hepatitis B-related nephropathy can effectively ensure the long-term survival of patients (5). In 2017, Jiang et al. (6). performed combined liver-pancreas-kidney transplantation for a patient with hepatitis B cirrhosis and IDDM using block transplantation and achieved good results. Combined pancreas-kidney transplantation can treat diabetes mellitus and diabetic renal failure. Compared with single kidney transplantation, combined pancreas-kidney transplantation can improve the quality of life, prevent or slow down the progression of diabetic complications, and prolong the survival time of recipients and transplanted kidneys (5). A study reported that 1,000 patients who underwent combined pancreas-kidney transplantation had 1-, 10-, and 20-year survival rates of 97, 80, and 58%, respectively, which were significantly better than those of type I diabetes patients with uremia who underwent single living kidney transplantation (403 cases) and single cadaveric kidney transplantation (697 cases) during the same period. The 10-year survival rate of diabetic nephropathy patients receiving hemodialysis was only 9.6–11.2% (7). According to the data of the United Network for Organ Sharing in 2016, the 5- and 10-year pancreas graft function rates after combined pancreas-kidney transplantation were 64 and 38%, respectively (8). Our patient had post-hepatitis B cirrhosis, hepatic insufficiency insulin, and chronic renal insufficiency accompanied with IDDM caused by chronic pancreatitis. After combined liver-kidney-pancreas transplantation, exogenous insulin was discontinued, liver and kidney functions and blood sugar levels returned to normal, the wound recovered well without a serious infection, and satisfactory results were achieved. Therefore, we believe that patients who require organ transplantation and have diabetes, whether kidney or liver transplantation, as long as insulin therapy is needed, should consider combined transplantation with pancreas transplantation. In this combined transplantation, the organs required for transplantation are mostly obtained from the same donor that have the same genetic background and the same antigenicity. Compared with organs from different donors, the number of donor specific antibodies produced will be reduced. The symptoms of diabetes can be simultaneously corrected with uremia and liver failure. Therefore, the long-term effect of combined liver-pancreas-kidney transplantation for patients with diabetes and liver and renal failures should be better than that of combined liver-kidney transplantation.

In multiple organ transplants, the risk of graft-vs.-host disease (GVHD) increases when the organs come from the same donor. GVHD is a kind of pathological damage caused by the antigen sensitization, proliferation and differentiation of the host's major histocompatibility complex (MHC) recognized by the donor's T lymphocytes contained in the graft, and then attacking the host's digestive tract, skin and other organs. GVHD mainly occurs in patients with small bowel transplantation and hematopoietic stem cell transplantation (9). GVHD is a serious complication after liver transplantation. Its incidence is about 2%, but the mortality rate is high (10). GVHD that occurs after solid organ transplantation mainly refers to: T lymphocytes present in the donor liver are activated after entering the recipient, and then cloned and expanded to attack the host tissues and organs. It is a serious complication after solid organ transplantation, with a mortality rate of more than 85% (11). The most commonly used diagnostic criteria for GVHD after solid organ transplantation are the three-point diagnostic criteria: patients with characteristic clinical symptoms and signs such as rash, fever, and diarrhea; the pathological examination and microbial culture of the involved and organs; evidence of donor lymphocytes found in peripheral blood or affected organs, such as the detection of donor lymphocyte DNA or HLA (12). Acute rejection occurred in this patient after the first transplant, and there was no evidence of GVHD.

### Long-Term Survival Analysis

A number of phenomena indicate that transplanted livers have immuno-protective effects. For example, although patients with liver transplantation do not undergo PRA and HLA examination, the incidence of acute rejection remains low, and acute-type rejection is less common in patients who undergo organ transplantation in combination with liver transplantation. From an immunological point of view, although the mechanism of liver immune preference is still not completely understood, there are some hypotheses that may explain it. One explanation is that a large number of passenger white blood cells in the transplanted liver form micro-chimerism with recipient cells and stably circulate and regenerate in the recipient, so that the recipient's immune system receives the donor antigen as if it were a self-antigen (13). Starzl et al. (14) confirmed the existence of peripheral micro-chimerism in liver transplant recipients, suggesting that chimerism plays an important role in inducing immune tolerance and may increase long-term graft survival. Shimonkevitz et al. (15) revealed that the removal of the chimeric state disrupts the state of tolerance and leads to rejection. The hypothesis that the liver can alleviate the rejection of other combined transplantation organs also suggests that the liver can reduce the incidence of antibody-mediated rejection by adsorbing lymphocyte-toxic antibodies *in vivo*. In addition, hepatocytes can express soluble class I antigens after transplantation, which is an immune response effect of the liver (16). Moreover, some studies have reported that the alleviation of rejection by the liver in other combined grafts may be because of larger liver volume and more antigens, resulting in low immune responsiveness; thus, subsequently, the regenerative ability of the liver may mask the slow rejection (17). This can also be explained by the fact that the multiorgan combined transplantation causes the recipient's immune system to bear an excessive immune load, resulting in a state in which the host's immune response to foreign antigens is ineffective. Other studies suggest that this immune protective effect may be related to the immune adsorption of donor-specific antibodies, which may be mediated by hepatocytes and FoxP3+ T cells and the cascade reaction after the activation of indoleamine-2,3-dioxygenase (18, 19). In our case, the first transplanted kidney was excised because of acute rejection, which proved that transplanted livers cannot exert sufficient immunoprotective effects on other grafts in all cases. The second successful kidney transplantation may be the result of the co-adsorbing of the transplanted liver and excised kidney.

### Selection of Immunological Factors and Immunosuppressive Programs

There were four mismatches in the first transplantion and six mismatches in the second transplantion. At that time, continuous renal replacement therapy (CRRT) was urgently needed by the patient, and the coagulation index of the patient would be seriously reduced after each dialysis, the bleeding tendency would be aggravated, and the maintenance of dialysis would be life-threatening. Therefore, although the matching results were not satisfactory, we still chose the second transplantation. In terms of epitope classification, A2, A9, A24, and A28 belong to the cross-reactive group (CREG) of A02C, B17, B15, B52, B50, and B46 belong to the CREG of B21C, DR4, DR9, and DR53 belong to the same CREG, so the increased donor antigen in the second transplantation does not significantly increase the potential risk of mismatch, which also can explain the good recovery of the patient.

Judging the degree of matching between donors and recipients and the producing of donor specific antibodies (DSA) by analyzing epitopes is a novel and effective method. HLA eplet matching can reflect the degree of incompatibility. Since this is a case in 2005, the HLA matching results at that time were detected by serological methods, and the results obtained were also serological results. However, the analysis of HLA eplet mismatches requires analysis with HLA high resolution results detected by sequence based typing (SBT). The existing serological HLA typing results cannot be converted to epitopes or eplets.

MMF was discontinued because of poor coagulation and serious bleeding in the early stage after the operation; moreover, continuous blood routine examination showed strong bone marrow suppression. Because of multiorgan transplantation, the antigenicity of the donor was relatively strong, and it was relatively easy to establish a balance with the recipient's immune system. Therefore, although the immunosuppressive regimen of FK506+Pred had been used for a long time, no clinical rejection occurred during the 14-year follow-up.

## Declaration

The case obeys Declaration of Istanbul. No executed prisoner donor was used. The donors had signed informed consents before donation. The documentation of the organ donation consent has been lost due to several relocations and personnel adjustments.

## Data Availability Statement

All datasets generated for this study are included in the article/supplementary material.

## Ethics Statement

Written informed consent was obtained from the individual(s) for the publication of any potentially identifiable images or data included in this article.

## Author Contributions

GZ, KD, and CM performed the operation. SY, ZL, and LY assisted in obtaining donors. MY was in charge of imaging diagnosis. XG, YZ, LW, and XY followed up patient and obtained data. JY, HW, and WQ concepted and supervised the study. All authors were involved in writing the paper and had final approval of the submitted and published versions.

## Conflict of Interest

The authors declare that the research was conducted in the absence of any commercial or financial relationships that could be construed as a potential conflict of interest.
